# Synaptic control of DNA methylation involves activity-dependent degradation of DNMT3A1 in the nucleus

**DOI:** 10.1038/s41386-020-0780-2

**Published:** 2020-07-29

**Authors:** Gonca Bayraktar, PingAn Yuanxiang, Alessandro D. Confettura, Guilherme M. Gomes, Syed A. Raza, Oliver Stork, Shoji Tajima, Isao Suetake, Anna Karpova, Ferah Yildirim, Michael R. Kreutz

**Affiliations:** 1 RG Neuroplasticity, Leibniz Institute for Neurobiology, Brenneckestr. 6, 39118 Magdeburg, Germany; 2 Center for Behavioral Brain Sciences, Otto von Guericke University, 39120 Magdeburg, Germany; 3 Department of Genetics and Molecular Neurobiology, Institute of Biology, Otto-von-Guericke University, Leipziger Str. 44, Haus 91, 39120 Magdeburg, Germany; 4 Institute for Protein Research, Osaka University, 3-2 Yamadaoka, Suita, 565-0871 Osaka Japan; 5 Department of Nutritional Sciences, Faculty of Nutritional Sciences, Nakamura Gakuen University, Fukuoka, Japan; 6 Laboratory of Organic Chemistry, Institute for Protein Research, Osaka University, Suita, Japan; 7 Center for Twin Research, Graduate School of Medicine, Osaka University, Suita, Japan; 8 NeuroCure Clinical Research Center & Department of Neuropsychiatry at Department of Psychiatry and Psychotherapy, Charité-Universitätsmedizin Berlin, Virchowweg 6, Charitéplatz 1, 10117 Berlin, Germany; 9 Leibniz Group ‘Dendritic Organelles and Synaptic Function’, ZMNH, 20251 Hamburg, Germany; 10 German Center for Neurodegenerative Diseases (DZNE), Magdeburg, Germany; 11 Present Address: UK Dementia Research Institute at the University of Cambridge, Island Research Building, Cambridge Biomedical Campus, University of Cambridge, Cambridge, CB2 0AH UK

**Keywords:** Neuroscience, Epigenetics and plasticity

## Abstract

DNA methylation is a crucial epigenetic mark for activity-dependent gene expression in neurons. Very little is known about how synaptic signals impact promoter methylation in neuronal nuclei. In this study we show that protein levels of the principal de novo DNA-methyltransferase in neurons, DNMT3A1, are tightly controlled by activation of N-methyl-D-aspartate receptors (NMDAR) containing the GluN2A subunit. Interestingly, synaptic NMDARs drive degradation of the methyltransferase in a neddylation-dependent manner. Inhibition of neddylation, the conjugation of the small ubiquitin-like protein NEDD8 to lysine residues, interrupts degradation of DNMT3A1. This results in deficits in promoter methylation of activity-dependent genes, as well as synaptic plasticity and memory formation. In turn, the underlying molecular pathway is triggered by the induction of synaptic plasticity and in response to object location learning. Collectively, the data show that plasticity-relevant signals from GluN2A-containing NMDARs control activity-dependent DNA-methylation involved in memory formation.

## Introduction

It is widely believed that rapid and reversible DNA methylation is essential for the stability of long-term memory but very little is known about how synaptic signals can induce changes in DNA methylation to elicit enduring alterations in plasticity-related gene expression [1–6]. In addition, aberrant DNA methylation has been implicated in neuropsychiatric diseases, including schizophrenia, bipolar, and major depressive disorders [1, 7, 8]. N-methyl-D-aspartate receptors (NMDAR) signaling to the nucleus is instrumental for learning and memory formation and is altered in schizophrenia as well as other neuropsychiatric disorders [9, 10]. However, a mechanistic link between NMDAR signaling and DNA methylation is currently missing.

Compelling evidence exists for learning-induced de novo DNA methylation with several studies showing the importance of active DNA methylation as well as demethylation particularly in the hippocampus during memory consolidation [1, 11–13]. One of the target genes is the brain-derived neurotrophic factor (BDNF), which undergoes promoter-specific DNA demethylation in the CA1 region of the hippocampus during memory consolidation [14]. The underlying signaling machinery in this process is also not well understood. It is fundamentally unclear how synaptic signals conveyed to the nucleus impact mechanisms of DNA methylation and demethylation of the *Bdnf* promoter. DNMT3A1 is the major de novo DNA methyltransferase in the brain and plays a documented role in activity-dependent DNA methylation [15, 16]. Impaired spatial learning and memory, as well as attenuated CA1 long-term potentiation (LTP), have been reported following a forebrain specific DNMT gene knockout in principal neurons [16, 17].

It is nowadays widely accepted that memory consolidation as well as synaptic plasticity not only rely on de novo protein synthesis but also protein degradation [18–20]. Proteasomal degradation of proteins in neurons has been studied mainly in the context of ubiquitylation and sumoylation, whereas neddylation, the attachment of the small ubiquitin-like peptide neural precursor cell-expressed developmentally down-regulated gene 8 (NEDD8) has not been thoroughly investigated. Here we show that activation of synaptic GluN2A-containing NMDARs drives the neddylation-dependent proteasomal degradation of the principal de novo DNA-methyltransferase in the adult brain DNMT3A1. Collectively, the data point to a mechanism that allows for the synaptic control of nuclear DNMT3A1 protein levels thereby creating a time window for reduced de novo DNA methylation at a subset of target genes. This signaling pathway highlights how synapse-to-nucleus signaling might directly impact on DNA methylation and memory consolidation.

## Methods

### Cell culture and drug treatments

Rat cortices and hippocampi were dissected from embryonic day 18 rats (Sprague Dawley). Cells were plated in a density of 30.000 cells per 18 mm coverslip, grown in 1 ml of neurobasal medium (NB, Gibco) supplemented with B27 medium. Primary neurons were kept in Neurobasal medium (NB/GIBCO/Life Technologies) supplemented with B27 (GIBCO/Life Technologies), L-glutamine (GIBCO/Life Technologies) and penicillin/streptomycin (PAA Laboratories, Pasching, Austria). On day 4 after plating cortical neurons were treated with 5 μM cytosine D-arabino-furanoside (Sigma-Aldrich) in order to prevent proliferation of non-neuronal cells.

Hippocampal neurons were treated for 10 min, 1 h, 3 h or 6 h at DIV 14–15 with the following drugs: Bicuculline methiodide (50 μM, Tocris, Bristol, UK), 4-aminopyridine (4AP, 2.5 mM, Sigma-Aldrich, St. Louis, MO), MG132 (30 μM, AG Scientific), lactacystin (15 μM, Sigma-Aldrich, St. Louis, MO), carfilzomib (100 nM, UBPBio), NVP-AAM007 (50 nM, Novartis), APV (20 μM, Calbiochem), ifenprodil (10 μM, Tocris, Bristol, UK), nifedipine (10 μM, Santa Cruz), KN93 (5 μM, Tocris, Bristol, UK) and MLN4924 (5 nM, Cayman Chemicals, Hamburg, Germany). Hippocampal neurons were treated for 3, 5 or 10 min with 100 μM N-methyl-D-aspartic acid (NMDA, Sigma-Aldrich, St. Louis, MO). Cortical neurons were seeded in T-75 flasks (Thermo Scientific, Rockford, USA). They were fed with the same neuron chow and proliferation of non-neuronal cells was arrested by the addition of 1 μM cytosine arabinoside (Sigma-Aldrich, St. Louis, MO) at DIV 5. Cortical neurons were treated with 1 μM tetrodotoxin (TTX, Alomone Labs, Jerusalem, Israel) for 12 h, media was washed-out and Bic/4AP were applied for 6 h at 21 DIV. HEK293T cells were cultured in DMEM media supported by fetal bovine serum. In all graphs depicting quantitative immunocytochemistry, the numbers for each experimental group indicate the number of analyzed neurons derived from at least two or three independent culture preparations, where from each culture preparation at least 2 culture wells are included in the study.

### Experimental animals

Neurons for primary cell cultures and slices for electrophysiology experiments were prepared from brains of Sprague Dawley (Janvier, France) or Wistar rats (Animal facilities of the Leibniz Institute of Neurobiology, Magdeburg, Germany). Male C57BL/6J (10–13 weeks old) mice were used for behavioral experiments (Charles River/Animal facilities of the Leibniz Institute of Neurobiology, Magdeburg, Germany). GluRε1 (GluN2A) knockout (KO) mice were obtained from RIKEN Japan (RBRC01813). Animals were housed in groups of up to 5 in individually ventilated cages (IVCs, Green line system, Tecniplast) under controlled environmental conditions (22 °C ± 2 °C, 55% ± 10% humidity, 12 h light/dark cycle, with lights on at 06:00). Food and water were available *ad libitum*. All procedures and animal care were consented and performed under established standards of the German federal state of Sachsen-Anhalt, Germany in agreement with the European Communities Council Directive (2010/63/EU) and approved by the local authorities of Sachsen-Anhalt/Germany/Regierungspräsidium Halle Sachsen-Anhalt/Germany.

### Methylation analysis

DNA from CA1 was extracted using Chargeswitch DNA extraction kit from Invitrogen (Carlsbad, CA, USA) according to the instructor’s manual. Methylation-sensitive restriction enzyme-dependent methylation analysis was performed using OneStep q-Methyl Kit (Zymo, Irvine, CA, USA) following the instructions given in the manual using 20 ng DNA for each sample. For methylated DNA immunoprecipitation (MeDIP) experiments, the extracted DNA was then subjected to fragmentation via a sonicator (Picoruptor, Diagenode, Seraing, Belgium) with 30 s on and 90 s off cycles using 1.5 ml Bioruptor microtubes. The efficiency of the sonication was established by running the fragmented DNA samples on agarose gels, which confirmed the accumulation of fragmented DNA to be mainly within a range of 200–600 bp. Afterward MeDIP experiments were carried out using the MagMeDIP kit (Diagenode, Seraing, Belgium) following the user guide with ~750 ng fragmented DNA as starting material. The methylation levels were assessed by qPCR and evaluated using the formula given in the user guide for both kits. The forward primer used in the OneStepqMethyl-Kit was 5′-TATGACAGCTCACGTCAAGG-3′ and reverse primer was 5′-CCTTCAGTGAGAAGCTCCAT-3′, containing three methylation-sensitive restriction enzyme sites. The forward primer used in the MeDIP-qPCR study for BDNF promoter IV was 5′-GCATGCAATGCCCTGGAACGG-3′ and the reverse primer was 5′-GAGGGCTCCACGCTGCCTTG-3′, and forward primer for BDNF promoter I 5′-TACCTGGCGACAGGGAAATC-3′ and reverse primer 5′-GCGCCCTAGCACAAAAAGTT-3′.

Percent methylation at single CpG sites in Bdnf IV promoter was determined using the bisulfite sequencing method. For this genomic DNA (gDNA) was extracted from 21 DIV cultured cortical neurons treated with Bic/4AP for 6 h or neurons that did not receive any treatment. The extraction was performed using Chargeswitch DNA extraction kit, as described above. 200 ng of gDNA was used for bisulfite conversion (EZ DNA methylation- gold, Zymo Research). To amplify the region of interest 1 to 2 μl of bisulfite-converted DNA was run in a PCR using specific primers spanning 19 CGs. Bisulfite sequencing primer sequences used to assess CpG methylation levels of the rat Bdnf promoter IV were: Forward 1–5′-TTTATAAAGTATGTAATGTTTTGGAA-3′, Forward 2–5′-AGTTAGTATGAAATTTTTTAGTTTTTGTT-3′, Reverse 1–5′-TTCAATAAAAAACTCCATTTAATCTA-3′, Reverse 2–5′-TATCAAAATAAACATCAAAACAACTAC-3′. The sequencing was performed on the MiSeq with a 600 cycle v3 kit, generating 300 base pair end reads. Mapping of the reads and methylation level extractions were performed using Bismark [21]. Average methylation rate of each CpG site was quantified using the methylKit R package [22]. The groups were compared using a Chi-square test and the correction applied was the false discovery rate (FDR) [23].

### Quantitative real time-PCR

14 DIV hippocampal neurons were treated with 50 μM bicuculline methiodide and 2.5 mM 4AP with or without 5 nM MLN4924 for 6 h. DMSO was used as a sham control. Following the treatment, neurons were immediately harvested in lysis buffer and total RNA was isolated (RNeasy plus mini kit, Qiagen, Valencia, CA, USA). 50 nanograms of RNA were reverse transcribed using random nonamers (Sigma-Aldrich, St. Louis, MO, USA) according to the manufacturer’s instructions (Sensiscript, Qiagen). BDNF exon IV and glyceraldehyde 3-phosphate dehydrogenase (GAPDH) mRNA (as a reference gene) were amplified using the iScript RT-PCR iQ SYBR Green Supermix (BIORAD, Hercules, CA, USA) in a real-time quantitative PCR (qPCR) detection system (LC480, Roche, Basel, Switzerland) using the following primers: *Bdnf* exon IV forward 5′-GCAGCTGCCTTGATGTTTAC-3′ and reverse 5′-CCGTGGACGTTTGCTTCTTTC-3′; *Dnmt3a1* forward 5′- CCCTCAGATCTGCTACCCAA-3′ and reverse 5′-TGCTCTGGAGGCTTCTGGTG-3′; *Dnmt3a2* forward 5′-CTCACACCTGAGCTGTACTGCAGAG-3′ and reverse 5′-CTCCACCTTCTGGGACTCCCCAGAG-3′; *Gapdh* forward 5′-TGCTGAGTATGTCGTGGAG-3′ and 5′-ACCCAGAAGACTGTGGATGG-3′; *Gapdh* reverse 5′- GTCTTCTGAGTGGCAGTGAT-3′ and 5′-CACATTGGGGGTAGGAACAC-3′. Each sample reaction was run in duplicate and Ct values of the reference genes from the samples were subjected to Grubbs’ outlier test (http://www.graphpad.com/quickcalcs/Grubbs1.cfm). The relative expression levels were analyzed using the 2−ΔΔCt method with normalization relative to GAPDH.

### Immunocytochemistry

Following drug treatment, cells were fixed either in methanol or in 4% paraformaldehyde (PFA) and immunostaining was performed with primary antibodies specific for the DNMT3AN-terminus, CUL4B (Proteintech, catalog #12916-1-AP), GFAP (Synaptic Systems, catalog #173011), HOMER1 (Synaptic Systems, catalog #160011), NEDD8 (Proteintech, catalog #16777-1-AP), MAP2 (Sigma-Aldrich, catalog #M4403), 5meCytosine (Calbiochem, catalog #MABE146, clone 33D3), Synaptophysin1 (Synaptic Systems, catalog #101004); secondary antibodies anti-rabbit/mouse-Alexa Fluor 488/568 linked (Molecular Probes Europe BV, Leiden, The Netherlands), anti-rabbit/mouse-Cy^TM^5-conjugated (Dianova, Hamburg, Germany) and DAPI (Sigma-Aldrich, catalog #D9564). Live immunostaining was performed for the detection of the surface expression of the GluN2A subunit of NMDARs. To this end, the GluN2A antibody (Alomone Labs, #AGC-002) was applied in cell media for 20 min, then cells were fixed and staining was performed as outlined above.

### Confocal laser scanning microscopy and image analysis

The SP5 CLSM system (Leica-Microsystems, Mannheim, Germany) equipped with Diode (405 nm) and Argon (488, 561, and 633 nm) laser lines was used for quantitative immunocytochemistry. Z-stack images of neurons were taken using the ×63 oil-immersion objective (Leica, Mannheim, Germany). Confocal images of triple-stained neurons were taken with Plan Apo ×63 oil NA 1.4 objective lenses. All images were acquired sequentially to avoid crosstalk between channels. The acquisition parameters were kept the same for all scans. Regions of interest (ROI) were drawn around nuclei, as delineated by DAPI staining. These ROI were then applied to a corresponding image of antibody staining from which the mean average intensity was collected to determine nuclear immunoreactivity levels using Image J software (NIH, Rasband, W.S., ImageJ, U. S. National Institutes of Health, Bethesda, Maryland, USA, http://imagej.nih.gov/ij/, 1997–2014).

The total number of dendritic spines per 20 μm secondary dendrites of the primary hippocampal neurons, which received 5 nM or 1 μM MLN4924 treatment at basal conditions or with synaptic stimulation using Bic/4AP were counted based on the Homer1 staining. The total synapse number per 20 μm secondary dendrites of the primary hippocampal neurons following Cul4B knockdown were counted based on the colocalization of Homer1 and Synaptophysin1. Surface expression of GluN2A was evaluated in primary hippocampal neurons following the knockdown of Cul4B.

### Fluorescent microscopy and image analysis

The number of dendrites crossing each circle was counted manually. Studies of GluN2A knockdown on hippocampal primary hippocampal neurons were also carried out using Zeiss Axio Imager A2 fluorescent microscope (Zeiss, Jena, Germany) with Cool Snap EZ camera (Visitron System) and MetaMorph Imaging software (MDS Analytical Technologies). Up to 3 coverslips were treated individually and processed per group. The same exposure time and intensity were taken for each coverslip among the different groups. Upon background subtraction using Fiji software, images of fluorescent positive puncta were measured along secondary dendrites, right after the branching point. The synaptic immunofluorescence intensities were assessed in a region of 400 nm × 400 nm square set by the mask in the channel for Homer1, a synaptic marker. The mask was created semi-automatically using Openview software. The intensities of the puncta positive for GluN2A were measured at points of co-localization with Homer1; values were normalized to mean of the control and plotted.

### Intrahippocampal injections

Mice were anesthetized with 5% isofluorane in O_2_/N_2_O mixture. Mice were placed in a stereotaxic frame (World Precision Instruments) and anesthesia was maintained with 1.5% isofluorane using gas anesthesia system (Rothacher Medical GmbH., Switzerland). After craniotomy, 10 μl NanoFil microsyringes (World Precision Instruments) containing 33G injection needles were lowered into the dorsal CA1 area under stereotactic guidance with the coordinates anterioposterior (AP) −2.0 mm, mediolateral (ML) ± 1.5 mm from Bregma and dorsoventral (DV) −0.14 mm from brain surface. Each animal received 1.5 μl/hemisphere bilateral infusion of drug (MLN4924) while (saline with respective volume of DMSO) as sham control, at an infusion rate of 0.5 μl/min.

### Overexpression and knockdown experiments

The overexpression experiments in HEK-293T cells were performed using the following constructs: C2-eGFP-hDNMT3A (Clontech, subcloned from pcDNA3/Myc-DNMT3A1, which was a gift from Arthur Riggs, Addgene plasmid #35521), C2-eGFP-hDNMT3A2 (Clontech, subcloned from pcDNA3/Myc-DNMT3A2, which was a gift from Arthur Riggs, Addgene plasmid #36941), pK-MYC-C3 (gift from V. Horejsi Lab, Prague, Czech Republic), pcDNA3-myc3-CUL4B (gift from Yue Xiong, Addgene plasmid #19922), pcDNA3-myc3-NEDD8 (gift from Yue Xiong, Addgene plasmid #19943), pcDNA3-HA-NEDD8 (gift from Edward Yeh, Addgene plasmid #18711), CUL1, CUL2, CUL3, CUL4A, CUL4B, CUL5 and CUL7 in pcDNA3-myc3 backbone (which were a gift from Yue Xiong, Addgene plasmid #19896, #19892, #19893, #19951, #19922, #19895, #20695 respectively). The vector for the expression of constitutively active CaMKIV with a nuclear localization signal (eGFP-CaMKIV-3xNLS) and dominant negative CaMKIV are were kindly provided by Dr. Gina Turrigiano (Waltham, USA) [24, 25]. The shRNA constructs to knock down human DNMT3A1/3A2 and scrambled controls were hDNMT3A shRNA, pSMP-DNMT3A1 and pSMP-Luc (a gift from George Daley, Addgene plasmid #36380, Addgene plasmid #36394, respectively). shRNA targeting, ratDNMT3A and scrambled control were cloned into pZ-off vector with the following sequences: 5′-CCCAAGGTCAAGGAGATCA-3′ and 5′-GCTTCGCGCCGTAGTCTTA-3′, respectively. shRNA targeting rat-Nedd8, human-Nedd8 and scrambled control were cloned into pSuper vector with the following sequences: 5′-GCGGCTCATCTACAGTGGCAA-3′, 5′-GAGGCTCATCTACAGTGGCAA-3′, 5′-CTTCGCGCCGTAGTCTTA-3′, respectively. The target shRNA sequence that efficiently knocked down rat-CaMKIV was 5′-CTAAGAAGCGGCTGACTAC-3′.

### Acute hippocampal slice preparation and electrophysiology

Hippocampi from 8 weeks old male Wistar rats (House strain, LIN, Magdeburg) were cut using a vibratome (LeicaVT1000S, Nussloch, Germany) into 350 μm thick slices. The hippocampal slices were incubated for 2 h in carbogenated (95% O_2_–5%CO_2_) artificial cerebrospinal fluid (ACSF, containing in mM: 110 NaCl, 2.5 KCl, 2.5 CaCl_2_·2H_2_O, 1.5 MgSO_4_·7H_2_O, 1.24 KH_2_PO_4_, 10 glucose, 27.4 NaHCO_3_, having a pH of 7.3) at room temperature. Then slices were transferred into a recording chamber (at 31 ± 1 °C). Field excitatory postsynaptic potentials (fEPSPs) were evoked by stimulation of CA1 Schaffer-collateral fibers with metal electrodes. fESPSs were recorded with ACSF filled glass capillary microelectrodes (3–5 MΩ) and amplified by an extracellular amplifier (EXT-02B, npi, Germany) and digitized at a sample frequency of 5 kHz by Digidata 1401 plus AD/DA converter (CED, England). Stimulation strength was adjusted to 30% – 40% of the maximum fEPSP-slope values. Single stimuli were applied every 30 s (at 0.0333 Hz) and were averaged every 3 min. After a 30 min stable baseline recording, MLN4924 was applied into the bath. 30 min later, late-long term potentiation (L-LTP) was induced by tetanization consisting of three 1 s stimulus trains at 100 Hz with a 6 min inter-train interval where the width of the single stimulus was 0.1 ms. MLN4924 was dissolved in DMSO and diluted in ACSF at final concentration (5 μM, or 50 nM) applied 30 min before the first tetanus. Data are represented as mean ± SEM.

### Tissue collection and analysis

After the LTP recordings, only the potentiated CA1 region of the Hippocampus was collected from the acute slices. The tissue was either subjected to RNA or DNA extraction for transcription or promoter methylation analysis, respectively, or for total protein extraction for western blotting. For the experiments investigating learning-dependent DNMT3A1 degradation, mice were sacrificed 3 or 6 h after training and the CA1 region of the hippocampus was dissected. Bilateral intrahippocampal MLN4924 (7.5 pmol/site) or saline infusions were performed immediately after training.

### Immunoprecipitation experiments

Endogenous IP experiments were performed from cultured rat cortical neurons. 50 μl dynabeads Protein G (ThermoFischer Scientific, Waltham, MA, USA) were blocked using albumin from chicken egg white (Sigma-Aldrich, St. Louis, MO, USA), while rocking for 30 min. Then, the dynabeads protein G were incubated with 2–3 μg of rabbit polyclonal anti-CUL4B antibody (Proteintech, catalog #20882-1-AP), while gently rotating for 2 h at 4 °C. Nuclear protein extracts were then incubated with antibody-bound-protein G dynabeads overnight at 4 °C. The next day, following several washes with ice-cold TBS-T, the samples were eluted using 4× Laemmli sample buffer.

Heterologous co-immunoprecipitation (het-coIP) experiments were performed in HEK-293T cells. Transfection of HEK-293T cells was performed with constructs pcDNA3-myc3-CUL4B, C2-eGFP-hDNMT3A, and pK-MYC-C3. 3 h prior to harvesting the cells, either the proteasome inhibitor MG132 (30 μM) or DMSO as sham control was applied to the cells. 24 h after transfection cells were harvested. In the het-coIP experiment in which the effect of neddylation was studied, transfections were performed using the constructs pcDNA3-myc3-CUL4B, C2-eGFP-hDNMT3A, pK-MYC-C3, and myc3-NEDD8. 24 h after transfection HEK-T cells were treated with NEDD8-activating enzyme inhibitor MLN4924 (94 nM) or DMSO as sham control for 24 h. MG132 application was done as mentioned above. Cell lysate extracts were incubated with the anti-GFP microbeads (MiltenyiBiotec GmbH, Gladbach, Germany) for 1 h. Eluted samples were run on SDS-PAGE.

### Western blot analysis

Total homogenates were prepared from mouse and rat brain or cultured cortical neurons by performing the lysis in 10 mM Tris/HCl pH 7.5, 0.5% TritonX, and a protease inhibitor cocktail (Roche, catalog #04693116001) containing TBS. 4× Laemmli buffer was added to produce a final dilution of 1.5×. Following 10 min-incubation at 95 °C, samples were ready for protein analysis. HEK-293T cells were harvested in TBS, which contained the protease inhibitor cocktail. Cells were lysed in 1% Triton-X containing lysis buffer; centrifuged for 30 min at 21,000 rpm and the supernatant fraction was collected. Protein estimation was performed either by amidoblack or BCA assay (Thermo Scientific). Western blots were then performed using 4–20% gradient polyacrylamide gels. The following antibodies were used in this study: DNMT3AN (1:2000) and DNMT3A-mid (1:2000), CUL4B (1:1000, Proteintech, catalog #12916-1-AP), NEDD8 (1:1000, Proteintech, catalog #16777-1-AP), FK1 (1:1000, Enzo, catalog #BML-PW8805), β-Actin (1:2000, Sigma-Aldrich, catalog #A5441), myc-tag (1:1000, Cell Signaling, catalog #2276), and horseradish peroxidase-coupled goat anti mouse/rabbit IgG-HRP linked secondary antibodies (1:20000, Jackson Immunoresearch Laboratories, catalog #115035003 and #111035003, respectively). Quantification of immunoblots was done with ImageJ software (NIH, Maryland, USA). Integrated density values were evaluated for the analysis. When necessary, data from individual experiments performed at different time points were normalized to respective controls and pooled together with other data sets.

### Object location memory

Object location memory was performed in a square arena (50 × 50 × 50 cm) under mild light conditions. Briefly, the task consisted of a habituation session, training and test. During habituation, animals were allowed to explore the empty arena for 20 min. Twenty-four hours later, a training session took place, where animals were free to explore a pair of similar objects (made of plastic mounting bricks), placed in the arena, for 20 min. A test session was performed 6 h after training, where one of the objects was placed in a new position, and again, animals were free to explore the two objects for 20 min. All three sessions were video-recorded and behavior was analyzed offline using ANY-maze software (ANY-mazeTM Video Tracking System, version 4.50, Stoelting Co. Wood Dale, USA). Exploration was recorded only when the animal touched or reached the object with the nose at a distance of less than 2 cm. The time mice spent exploring the objects was recorded, and the discrimination index was then calculated, taking into account the difference of time spent exploring the new and familiar position ([(Tnovel – Tfamiliar)/(Tnovel + Tfamiliar)] × 100). An experimenter blind to treatment conducted the experiment and analyzed the data. Chambers and objects were thoroughly cleaned with 10% ethanol before and after each animal was tested.

### Statistical analysis

The data were analyzed by one-way ANOVA and unpaired one/two-tailed Student’s *t*-test. Two-way ANOVA followed by Bonferroni’s post-hoc test was employed to compare means from multiple groups. Quantitative real-time PCR data were subjected to Grubbs’ outlier test and analyzed by either unpaired two-tailed Student’s *t*-test or two-way ANOVA, which was followed by Bonferroni’s post hoc test, where applicable. The Mann Whitney *U*-test was used to compare the Averaged field potentials (300 – 360 min) between two groups of differentially treated slices. Error bars present S.E.M. except when otherwise stated. Statistical analyses were performed in GraphPad (GraphPad Software, Inc., La Jolla, USA).

## Results

### Synaptic activity controls levels of DNMT3A1 in neuronal nuclei

**Fig. 1:**
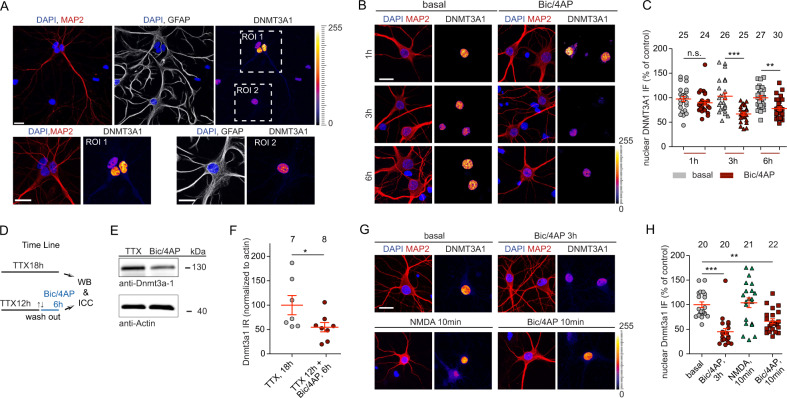
Synaptic activity regulates DNMT3A1 protein levels in neurons. **a** Nuclear DNMT3A1 immunofluorescence is prominent in MAP2-positive neurons but much less in GFAP-positive astrocytes at 15 DIV hippocampal cultures. Scale bars are 20 μm. **b**, **c** Downregulation of DNMT3A1 protein levels in 14–15 DIV hippocampal primary neurons following treatment with Bic/4AP for 1 h, 3 h or 6 h as evidenced by quantitative immunocytochemistry. Scale bar is 20 μm. Unpaired Student’s *t*-test **p < 0.01, ***p < 0.001. **d**–**f** DNMT3a1 protein levels are decreased following the removal of tonic inhibition in DIV 21 cortical neurons. β-Actin was used as an internal control for normalization. Student’s *t*-test *p < 0.05. **g**, **h** Hippocampal neurons were treated with Bic/4AP for 10 min or 3 h. Media from 10 min-long treated neurons was washed out and cells were kept for 3 h before fixation. Both treatments were equally effective to reduce nuclear DNMT3A1 immunofluorescence. Application of 100 μM NMDA for 10 min had no effect. Unpaired Student’s *t*-test **p < 0.01, ***p < 0.001. Error bars present S.E.M. Sample numbers for each experimental group indicate neurons from three different culture preparations.

DNMT3A1 is the major de novo DNA methyltransferase expressed in the adult brain [15]. In addressing the cellular localization of this enzyme using an antibody that recognizes an N-terminal region [26] specific for DNMT3A1 (Fig. S1A–D), we found a prominent nuclear localization in hippocampal primary neurons and much fainter staining hardly above background in astrocytes (Fig. 1a), indicating that DNMT3A1 is mainly expressed in neurons. This finding prompted us to ask next whether synaptic activity might regulate nuclear DNMT3A1 protein levels. When we induced burst firing of excitatory synapses in hippocampal primary neurons with the GABA-A receptor antagonist bicuculline (Bic) and the potassium channel blocker 4-amino-pyridine (4AP), we observed a prominent reduction in the nuclear immunofluorescence of DNMT3A1 (Fig. 1b, c), a finding that was confirmed by quantitative immunoblot analysis of cell lysates from cortical primary neurons (Fig. 1d–f). Enhancing excitatory activity for 10 min with Bic/4AP was sufficient to reduce DNMT3A1 immunofluorescence for 3 h following stimulation (Fig. 1g, h), while excitotoxicity, elicited with bath application of 100 µM NMDA for 10 min, a protocol that results in extrasynaptic NMDAR activation and delayed cell death, had no effect [27]. Of note, the reduction of DNMT3A1 protein levels lasted at least for 24 h and returned back to baseline expression only after 72 h (Fig. S1E–J).

### Synaptic GluN2A-containing NMDARs drive the degradation of nuclear DNMT3A1

**Fig. 2:**
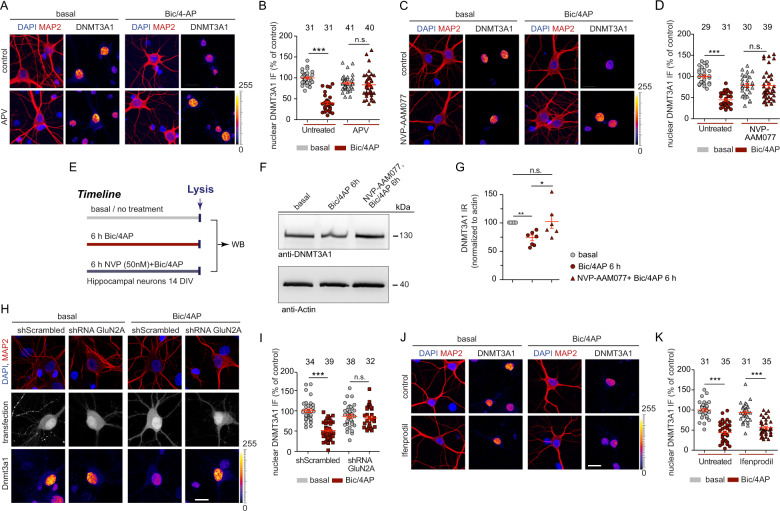
GluN2A-dependent NMDAR signaling regulates nuclear DNMT3A1 protein levels in 14–15 DIV hippocampal primary neurons. **a**, **b** Treatment with the NMDAR antagonist APV (20 μM) prevented the reduction in the nuclear levels of DNMT3A1 following synaptic stimulation by Bic/4AP for 6 h. **c**, **d** Treatment with the GluN2A inhibitor NVP-AAM077 (50 nM) prevented the reduction in the nuclear levels of DNMT3A1 following synaptic stimulation by Bic/4AP for 6 h. Two-way ANOVA followed by Bonferroni’s post hoc test. **e** Timeline view of hippocampal neuron treatment by Bic/4AP for 6 h with or without NVP-AAM077. **f**, **g** Quantitative immunoblotting revealed the inhibition of DNMT3A1 degradation by the use of NVP-AAM077. One-sample t-test is performed while the hypothetical value is set to 100. **h**, **i** shRNA-based knockdown of GluN2A in the presence of synaptic stimulation by Bic/4AP for 6 h prevented the DNMT3A1 degradation. **j**, **k** Treatment with the GluN2B subunit inhibitor ifenprodil (10 μM) in the presence of synaptic stimulation with Bic/4AP for 6 h did not prevent the reduction in the nuclear levels of Dnmt3A1. Scale bars, 20 μm. Two-way ANOVA followed by Bonferroni’s post hoc test. ***p < 0.001, n.s. not significant. Error bars present S.E.M. Sample numbers for each experimental group indicate neurons from three different culture preparations.

The activity-dependent degradation of DNMT3A1 was blocked in the presence of the competitive NMDAR antagonist 2-amino-5-phosphonopentanoic acid (APV) (Fig. 2a, b). Interestingly, the application of the antagonist NVP-AAM077, which mainly targets di-heteromeric GluN2A-containing NMDARs [28] in low doses, completely prevented degradation as evidenced by quantitative immunocytochemistry (Fig. 2c, d) and immunoblotting (Fig. 2e, f). In addition, shRNA-induced protein knockdown (Fig. S2A, B) of GluN2A confirmed a requirement for NMDARs specifically containing this subunit to elicit degradation of DNMT3A1 (Fig. 2h, i), whereas application of the GluN2B antagonist ifenprodil had no effect (Fig. 2j, k). Co-application of the L-type Ca^2+^-channel blocker nifedipine (Fig. S2C, D) or the CaMKII and CaMKIV inhibitor KN93 to the stimulation buffer also hindered nuclear DNMT3A1 degradation (Fig. S2E, F). Likewise, CaMKIV shRNA knockdown clearly reduced DNMT3A1 degradation in response to enhanced synaptic activity (Fig. S2G–I). Similarly, nuclear overexpression of a dominant negative form of CaMKIV reduced activity-dependent degradation, whereas constitutively active CaMKIV [19, 20] had the opposite effect (Fig. S2J–L). Taken together, these experiments indicate that brief activation of synaptic GluN2A-containing NMDARs is a potent stimulus to control DNMT3A1 protein levels in the nucleus and that synaptic control probably involves the induction of backpropagating dendritic action potentials and nuclear calcium waves which in turn activate CaMKIV, upstream of DNTM3A1 downregulation.

### Proteasomal degradation of DNMT3A1 requires neddylation

We next examined which mechanisms might contribute to DNMT3A1 downregulation and found that the proteasome inhibitors MG132, lactacystin or carfilzomib, which all operate via different mechanisms [29–31], completely abolished the effect of GluN2A stimulation (Fig. S3A–F). Since we found no concomitant alteration of *Dnmt3a1* mRNA levels (Fig. S3G-I), these data suggest that proteasomal degradation controls the protein levels of the enzyme in an activity-dependent manner.

**Fig. 3:**
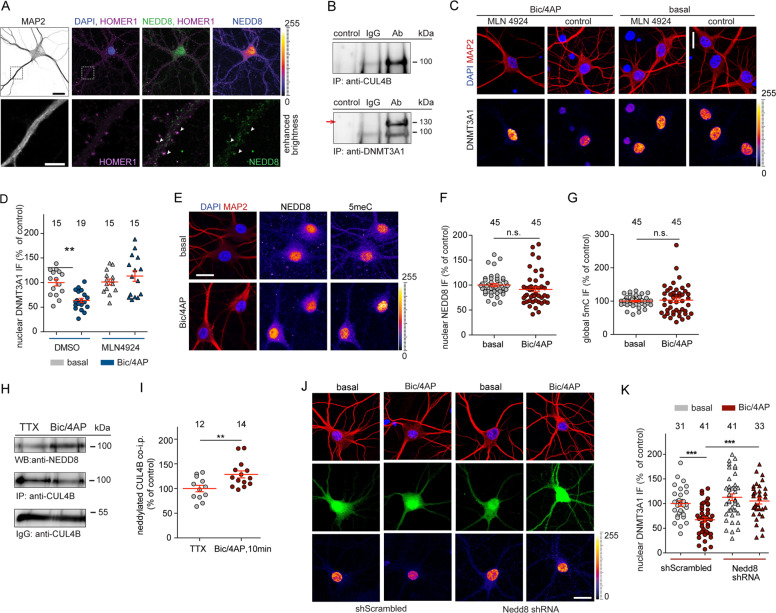
DNMT3A1 degradation is a neddylation-dependent process. **a** NEDD8 is abundantly expressed in the nucleus of 16 DIV hippocampal neurons whereas weaker staining was observed at synapses. Scale bar 20 μm (top panel), 5 μm (lower panel). **b** Immunoprecipitation of CUL4B protein from nuclear extracts of cortical primary neurons (top panel) results in co-precipitation of DNMT3A1 (lower panel, indicated with an arrow). **c**, **d** Hippocampal primary neurons were treated with Bic/4AP for 6 h in the presence or absence of MLN4924 (5 nM). Scale bars, 20 μm. Quantitative immunocytochemistry revealed that blocking neddylation prevents DNMT3A1 degradation. **e** Representative immunofluorescence images of nuclear NEDD8 and total cytosine methylation (5meC) at basal conditions or after treatment with Bic/4AP for 6 h. Scale bar is 20 μm. **f**, **g** Upon synaptic activation nuclear NEDD8 and 5meC levels remained unchanged. Unpaired Student’s *t*-test. n.s. not significant. **h**, **i** CUL4B was immunoprecipitated from nuclear extracts of primary cortical neurons and neddylated CUL4B was quantified. Following 10 min of synaptic stimulation, the amount of neddylated CUL4B was increased. Unpaired Student’s *t*-test **p < 0.01. **j**, **k** shRNA knockdown of NEDD8 in hippocampal primary neurons reduced DNMT3A1 degradation following 10 min-long Bic/4AP treatment and fixation of cells 3 h after washout of the drug-containing-media. Two-way ANOVA followed by Bonferroni’s post-hoc test. ***p < 0.001, scale bar, 20 μm. Error bars present S.E.M. Sample numbers for each experimental group indicate neurons from three different culture preparations.

Potential mediators of DNMT3A1 degradation are members of the family of Cullin proteins [32]. Cullin family members combine with RING proteins to form Cullin-RING E3 ubiquitin ligases [33] and neddylation is a prerequisite for their activation in the nucleus. Neddylation has been studied only recently in neurons [34, 35] and the subcellular distribution of NEDD8 in neurons has not been determined yet. We found that NEDD8 is abundantly localized in the nucleus of hippocampal primary neurons, whereas immunofluorescence intensity was much weaker at synapses (Fig. 3a). We observed heterologous co-immunoprecipitation of DNMT3A1 with CUL4B, CUL1, CUL3, CUL4, CUL7, but not with CUL2 and CUL5 from HEK293T cell lysates (Fig. S4A). In addition, we found that poly-ubiquitination of DNMT3A1 was elevated following forced expression of CUL4B in HEK293T cells (Fig. S4B, D), which was chosen as an example because of its abundance in the brain. Conversely, shRNA-based CUL4B protein knockdown resulted in reduced DNMT3A1 poly-ubiquitination (Fig. S4E). The neddylation inhibitor MLN4924 selectively inhibits the NEDD8-Activating-Enzyme (NAE) at very low concentrations [36]. Accordingly, poly-ubiquitination of immunoprecipitated DNMT3A1 was reduced in cells that were subjected to MLN4924 treatment (Fig. S4F). Moreover, neddylated CUL4B was co-immunoprecipitated with DNMT3A1 (Fig. S4G).

Following this, we asked whether neddylation of Cullins might be involved in controlling activity-dependent DNMT3A1 proteasomal degradation in neurons. With co-immunoprecipitation experiments on endogenous proteins extracted from cortical primary neurons using a CUL4B specific antibody as *pars pro toto* we revealed that neuronal CUL4B and DNMT3A1 might be in one complex in vivo (Fig. 3b). Acute treatment of hippocampal primary neurons with concentrations of MLN4924 as low as 5 nM prevented DNMT3A1 degradation (Fig. 3c, d). Of note, acute treatment of primary neurons for 6 h with either a low (5 nM) or even a high dose of MLN4924 (1 µM) did not alter the total number of spines (Fig. S5A, B) as it was reported previously with a higher concentration and long-term treatment [34, 35]. Moreover, we observed that nuclear NEDD8 staining intensity was not altered following Bic/4AP treatment (Fig. 3e, f), which excludes the possibility that activity-dependent nucleocytoplasmic shuttling of NEDD8 contributes to DNMT3A1 degradation. In addition, 5meC immunocytochemistry revealed that no gross quantitative alterations in global DNA methylation occur following sustained stimulation of synaptic GluN2A-containing NMDARs (Fig. 3e, g). However, enhanced synaptic activity resulted in rapidly increased neddylation of CUL4B (Fig. 3h, i), whereas shRNA knockdown of NEDD8 (Fig. S5C–E) prevented activity-dependent degradation of DNMT3A1 (Fig. 3j, k). Collectively, the biochemical, pharmacological and shRNA knockdown experiments show that synaptic activation of GluN2A-containing NMDARs will drive neddylation of Cullin-ligases in the nucleus, which is a prerequisite for DNMT3A1 degradation.

### Synaptic plasticity inducing stimuli elicit DNMT3A1 degradation in a GluN2A-dependent manner

**Fig. 4:**
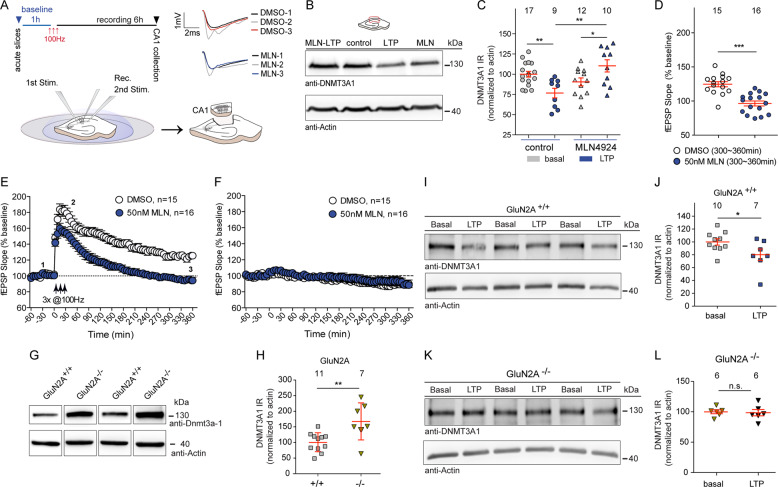
The NEDD8 inhibitor MLN4924 impairs LTP in rat hippocampal slices and degradation of DNMT3A1 upon LTP induction is absent in GluN2A knock out mice. **a** Schematic presentation of LTP induction by high-frequency stimulation in acute CA1 hippocampal slices. Only the potentiated CA1 region was dissected from individual hippocampal slices 6 h following LTP recordings. Representative traces from recordings at the time points indicated under control and treatment conditions. **b**, **c** DNMT3A1 levels are reduced 6 h after LTP induction and neddylation inhibition prevented the degradation of Dnmt3A1. β-Actin was used as an internal control for normalization. Two-way ANOVA followed by Bonferroni’s post hoc test. **p < 0.01, *p < 0.05. **d** Averaged fEPSP slopes of the last hour following LTP induction showed significantly reduced LTP in MLN4924 (50 nM) treated slices in comparison to slices treated by DMSO. Mann–Whitney *U*-Test ***p < 0.001. **e** Application of MLN4924 for 6 h induces significantly impaired LTP as compared to controls. *p < 0.05 and **p < 0.01. **f** Baseline recordings revealed no alterations. **g**, **h** GluN2A knockout (KO) mice show higher DNMT3A1 protein levels in the CA1 region of the hippocampus compared to the age-matched wild type (WT) control mice. unpaired Student’s *t*-test **p < 0.01. **i**–**l** Quantitative immunoblotting either from the CA1 of WT (**i**, **j**) or GluN2A KO (**k**, **l**) mice revealed no reduction in the GluN2A KO mice following 3 h of LTP recordings unlike reduction seen in the WT control mice tissue. Unpaired Student’s *t*-test. *p < 0.05, n.s. not significant. Error bars present S.E.M. Sample numbers for each experimental group indicate potentiated pooled CA1 slices from at least three different recordings or mice used per group.

We then addressed whether induction of NMDAR-dependent LTP, a form of plasticity that is considered to be a cellular model of learning and memory, impacts nuclear DNMT3A1 protein levels. When we induced LTP in the hippocampus with high-frequency stimulation of Schaffer-collaterals (Fig. 4a), we found a significant downregulation of DNMT3A1 protein levels within 6 h in the potentiated CA1 region following tetanization of the slices (Fig. 4b, c). Moreover, the expression of late-LTP is neddylation-sensitive and field excitatory postsynaptic potentiation slope values returned to baseline within three hours when the slices were treated with the NEDD8 inhibitor MLN4924 like reported previously [37] (Fig. 4d–f).

We subsequently investigated whether following the induction of LTP signaling of GluN2A-containing NMDARs to the nucleus is crucial in neddylation-dependent degradation of DNMT3A1. Interestingly, DNMT3A1 protein levels were already clearly higher in hippocampal tissue homogenates of GluN2A knockout mice as compared to wild-type controls (Fig. 4g, h). GluN2A knockout mice show reportedly impaired hippocampal LTP [38] but a stronger tetanic stimulation rescues this impairment and the saturation level of LTP remains unaltered [39]. We could replicate these published findings (Fig. S6A, C) and found that despite the induction of LTP with a stronger protocol no reduction in nuclear DNMT3A1 protein levels like in wild-type mice was detectable (Fig. 4i–l). Notably, while we observed a negative correlation between the strength of LTP and the magnitude of DNMT3A1 degradation in wild-type control animals (Fig. S7D) no correlation was seen in GluN2A -/- mice despite recovered LTP. Thus, GluN2A signaling in synaptic plasticity and not the induction of LTP as such is instrumental in controlling nuclear protein levels of DNMT3A1.

### Neddylation facilitates Bdnf gene expression

In the adult brain, BDNF has principal functions in synaptic plasticity, learning, and memory [40]. *Bdnf* gene expression is controlled by eight promoters [41] and among those, particularly promoter IV activity is strongly stimulated by the calcium influx through synaptic NMDARs [14, 42]. DNA methylation of the *Bdnf* *IV* promoter has been studied previously also in the context of neuropsychiatric disorders [43, 44]. We could replicate these findings and profiled the changes in DNA methylation levels following neuronal activity by the 6 h-long Bic/4AP treatment with bisulfite sequencing that cover the 19 CGs in *Bdnf IV* gene promoter (Fig. S7A). We, therefore, chose *Bdnf* *IV* gene expression to test whether neddylation and activity-dependent degradation of DNMT3A1 might impact DNA methylation of promoters of plasticity-related genes and corresponding gene expression. Quantitative real-time PCR experiments first revealed that *Bdnf IV* mRNA expression is increased by enhanced synaptic activity in primary hippocampal neurons and this increase in transcript levels was significantly lower in the presence of MLN4924 (Fig. S7B). Comparable results were obtained in acute hippocampal slices following high-frequency stimulation of Schaffer-collaterals (Fig. S7C) and importantly, the LTP-induced increase in *Bdnf IV* transcript levels was reduced in the presence of the NEDD8-inhibitor (Fig. S7C).

We next addressed whether increased *Bdnf IV* mRNA production was correlated with the demethylation of the *Bdnf IV* promoter in acute slices following LTP induction, as predicted by the degradation of DNMT3A1. First, methylation specific restriction enzyme analysis was performed using primers that span the *Bdnf IV* promoter sequence possessing three different restriction sites (Fig. S7G). Tetanized CA1 samples revealed a reduction in *Bdnf IV* promoter methylation, whereas increased promoter methylation was observed for the group that received high-frequency stimulation while being treated with MLN4924 (Fig. S7D). A subsequent series of MeDIP-qPCR experiments were performed using tetanized CA1 tissue samples following the induction of LTP and confirmed the change in promoter methylation. Less amplicons were generated with primers targeting the *Bdnf IV* promoter that cover multiple cytosine residues (Fig. S7H) known to regulate mRNA expression (Fig. S7E). More amplicons were detected in MLN4924-treated slices following the induction of LTP (Fig. S7E), indicating increased promoter methylation. Among the different *Bdnf* promoters that were investigated, activity-dependent DNA methylation is particularly prominent for promoter IV, whereas promoter I methylation was not altered following either LTP induction or NEDD8 inhibition with MLN4924 (Fig. S7F).

### DNMT3A1 is degraded in the hippocampus as a result of learning

**Fig. 5:**
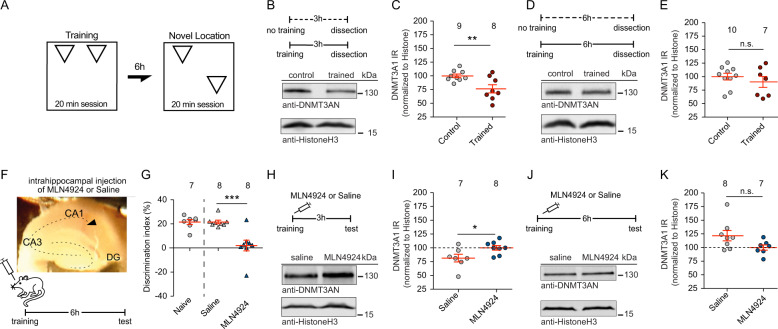
Inhibition of neddylation in CA1 impairs object location memory and the learning-induced degradation of DNMT3A1. **a** Schematic representation of the experimental protocol for object location memory. **b**, **c** DNMT3A1 protein levels are reduced 3 h after training. Unpaired Student’s *t*-test *p < 0.05. **d**, **e** DNMT3A1 protein levels are not changed in trained mice 6 h after training. Histone was used as a loading control for normalization. Unpaired Student’s *t*-test n.s. = not significant. **f** Injection timeline and representative image of injection site in CA1. **g** Intra-hippocampal infusion of MLN4924 reduces the discrimination index of animals tested in the object location memory test. Unpaired Student’s *t*-test with Welch correction ***p < 0.001. **h**, **i** Learning induced reduction of DNMT3A1 protein levels in trained, but not MLN4924 injected mice 3 h after training. Unpaired two-tailed Student’s *t*-test *p < 0.01. **j**, **k** DNMT3A1 protein levels were not altered in trained MLN4924 injected or control mice 6 h after training. Histone was used as a loading control for normalization. Data are represented as mean ± S.E.M. Unpaired Student’s *t*-test *p < 0.05. Sample numbers for each experimental group indicate mice used per group.

In the final set of experiments, we investigated whether DNMT3A1 degradation occurs in vivo as a result of CA1-dependent learning and whether this degradation and memory formation is neddylation-sensitive. Formation of a memory for the spatial location of objects in an open field (Fig. 5a) requires synaptic activity of CA1 neurons [45, 46] and is responsive to changes in the expression of BDNF [47, 48]. We observed that DNMT3A1 protein levels were reduced in mice for three hours following training (Fig. 5b, c). DNMT3A1 protein levels returned to control values within 6 h, which may reflect less intense synaptic activity as compared to tetanization of slices by high-frequency stimulation (Fig. 5d, e). Bilateral intrahippocampal infusion of MLN4924 into CA1 immediately after object location learning (Fig. 5f) resulted in a disturbance of object location memory, as indicated by profoundly reduced discrimination of novel and familiar object locations when compared to mice that received vehicle infusion (Fig. 5g). Interestingly, the learning impairment in MLN4924 treated mice was associated with the prevention of DNMT3A1 degradation (Fig. 5h, i). Protein levels were significantly higher in mice injected with MLN4924 three hours after training compared to vehicle-injected mice (Fig. 5h, i). Six hours following training DNMT3A1 protein levels were no longer different between treatment groups and returned to baseline levels (Fig. 5j, k).

## Discussion

Compelling evidence exists for the necessity of active DNA methylation as well as demethylation during memory consolidation in the hippocampus [1, 2, 11–13]. However, the underlying signaling machinery is not understood and it is essentially unclear how synaptic signals conveyed to the nucleus impact DNA methylation and demethylation. Here, we show that activation of synaptic GluN2A-containing NMDARs drives the neddylation-dependent proteasomal degradation of DNMT3A1, the principal de novo DNA-methyltransferase in the adult brain.

The finding that signals deriving from synaptic GluN2A-containing NMDARs evoke degradation of DNMT3A1 raises several questions about the underlying mechanism of long-distance signaling and the rationale behind it. Since GluN2A-containing NMDARs are in contrast to those containing GluN2B preferentially found at synaptic sites [49], it is possible if not likely that steep and fast synaptic Ca^2+^-influx through these receptors is necessary to elicit nuclear Ca^2+^-responses that in turn enhance neddylation of Cullins in a CaMKIV-dependent manner. Neddylation as such has not been investigated in any detail in neurons yet and not much information is currently available on how NAE activity itself is regulated. The present study, therefore, provides first evidence that an NMDAR-derived synaptic calcium signal is coupled to neddylation of Cullins in the nucleus. Two previous reports have shown that blocking neddylation for extended periods of time (in contrast to the administration regime in the present study) leads to reductions in spine size and impairment of synapse maturation in neurons [34, 35]. In addition, neddylation alters synapse function and morphology by directly modifying one of the major synaptic scaffolding proteins PSD95 [35]. We found that NEDD8 is most abundant in neuronal nuclei and it is tempting to speculate that activity-dependent neddylation might reduce the protein levels not only of DNMT3A1 but also of other nuclear epigenetic modifiers, which contribute to object location memory. Along these lines the contribution of neddylation to object location memory seems to be substantial, considering the near-complete removal of the discrimination upon the inhibition of neddylation. This is, however, not reflected in the extent of reduction in the levels of DNMT3A1 induced by the behavioral training. Moreover, neddylation, like other post-translational modifications, is reversible, which adds potentially another level of regulation. In addition, different efficiencies of proteasomal degradation in neuronal sub-compartments, the necessity for the integration of signaling pathways in the nucleus as well as complex formation and potential nuclear export of ubiquitinated DNMT3A1 may account for the relatively slow decline.

The proposed long-distance signaling pathway can provide a potential link between different observations. Converging evidence suggests that NMDAR function in the dorsal CA1 area is critical for novel object location memory [45, 46] and increased BDNF expression in the hippocampal CA1 region supports object location learning [47, 48]. We have, therefore, chosen *Bdnf* as a paradigmatic example for our studies, which is one of the target genes that undergoes promoter-specific DNA demethylation in the CA1 region of the hippocampus during memory consolidation [14] and impaired spatial learning and memory, as well as attenuated CA1-LTP, have been reported following a forebrain specific *Dnmt1* and *-3* gene knockout in principal neurons [16, 17]. Aberrant DNA methylation has been implicated in a plethora of studies on neuropsychiatric diseases including schizophrenia, bipolar, and major depressive disorders [1, 7, 8]. One of the hallmarks of schizophrenia is a downregulation of BDNF expression that is associated with the enrichment of 5-methylcytosine at gene regulatory domains within the *Bdnf* promoter [50]. Moreover, elevated hippocampal DNMT3A expression has been reported in the postmortem brain of schizophrenia patients [51].

Collectively our data point to a mechanism that allows for the synaptic control of DNMT3A1 levels and thereby creates a time window for reduced de novo DNA methylation at a subset of target genes. DNMT3A1-mediated methylation has been largely associated with the silencing of promoters, which would in turn attenuate activity-dependent gene expression. A shorter splice isoform, DNMT3A2, was shown to associate with transcription of plasticity-relevant genes presumably via methylation of CpG islands in their promoter and coding regions [52, 53]. *Dnmt3a2* is an immediate early gene that is identical to *Dnmt3a1* except that it lacks the sequence encoding the N-terminal 219 amino acids of the enzyme, which encompasses the epitope of the antibody that was used in the current study. Intriguingly, we found increased expression of *Dnmt3a2* mRNA (Fig. S3I) and downregulation of DNMT3A1 is induced by the same stimulus, i.e. activation of synaptic GluN2A-containing NMDARs. It is currently unknown whether *Dnmt3a2* mRNA will be immediately translated, as expected for an immediate early gene. In this case, it may not replace DNMT3A1 but independently facilitate activity-dependent gene expression.

## Funding and disclosure

The authors have no conflict of interest to declare. Supported by grants from the Deutsche Forschungsgemeinschaft (DFG) (Kr 1879/9-1/FOR 2419, Kr1879/5-1/6-1/10-1; CRC 779 TPB8; Research Training Group 2413 SynAGE), BMBF ‘Energi’ FKZ: 01GQ1421B, The EU Joint Programme—Neurodegenerative Disease Research (JPND) project STAD (01ED1613) and Leibniz Foundation SAW to MRK. CRC 779 TPB8 to AK. Deutsche Forschungsgemeinschaft SFB 779 TPB5 to OS. Grants-in-Aid for Scientific Research B from the Japan Society for the Promotion of Science to ST; Deutsche Forschungsgemeinschaft EXC 257/2 to FY. GMG was supported by a CAPES-Alexander von Humboldt Research Fellowship (1756/14-1). Open access funding provided by Projekt DEAL.

## Supplementary information

Supplement

## References

[1] Bayraktar G, Kreutz MR (2018). Neuronal DNA methyltransferases: epigenetic mediators between synaptic activity and gene expression?. Neuroscientist.

[2] Bayraktar G, Kreutz MR (2018). The role of activity-dependent DNA demethylation in the adult brain and in neurological disorders. Front Mol Neurosci.

[3] Campbell RR, Wood MA (2019). How the epigenome integrates information and reshapes the synapse. Nat Rev Neurosci.

[4] Day JJ, Sweatt JD (2010). DNA methylation and memory formation. Nat Neurosci.

[5] Guo JU, Ma DK, Mo H, Ball MP, Jang MH, Bonaguidi MA (2011). Neuronal activity modifies the DNA methylation landscape in the adult brain. Nat Neurosci.

[6] Miller CA, Sweatt JD (2007). Covalent modification of DNA regulates memory formation. Neuron.

[7] Mill J, Tang T, Kaminsky Z, Khare T, Yazdanpanah S, Bouchard L (2008). Epigenomic profiling reveals DNA-methylation changes associated with major psychosis. Am J Hum Genet.

[8] Murgatroyd C, Patchev AV, Wu Y, Micale V, Bockmuhl Y, Fischer D (2009). Dynamic DNA methylation programs persistent adverse effects of early-life stress. Nat Neurosci.

[9] Paoletti P, Bellone C, Zhou Q (2013). NMDA receptor subunit diversity: impact on receptor properties, synaptic plasticity and disease. Nat Rev Neurosci.

[10] Zhou Q, Sheng M (2013). NMDA receptors in nervous system diseases. Neuropharmacology.

[11] Kaas GA, Zhong C, Eason DE, Ross DL, Vachhani RV, Ming GL (2013). TET1 controls CNS 5-methylcytosine hydroxylation, active DNA demethylation, gene transcription, and memory formation. Neuron.

[12] Oliveira AM (2016). DNA methylation: a permissive mark in memory formation and maintenance. Learn Mem.

[13] Rudenko A, Dawlaty MM, Seo J, Cheng AW, Meng J, Le T (2013). Tet1 is critical for neuronal activity-regulated gene expression and memory extinction. Neuron.

[14] Lubin FD, Roth TL, Sweatt JD (2008). Epigenetic regulation of BDNF gene transcription in the consolidation of fear memory. J Neurosci.

[15] Feng J, Chang H, Li E, Fan G (2005). Dynamic expression of de novo DNA methyltransferases Dnmt3a and Dnmt3b in the central nervous system. J Neurosci Res.

[16] Feng J, Zhou Y, Campbell SL, Le T, Li E, Sweatt JD (2010). Dnmt1 and Dnmt3a maintain DNA methylation and regulate synaptic function in adult forebrain neurons. Nat Neurosci.

[17] Morris MJ, Adachi M, Na ES, Monteggia LM (2014). Selective role for DNMT3a in learning and memory. Neurobiol Learn Mem.

[18] Dong C, Bach SV, Haynes KA, Hegde AN (2014). Proteasome modulates positive and negative translational regulators in long-term synaptic plasticity. J Neurosci.

[19] Jarome TJ, Helmstetter FJ (2013). The ubiquitin-proteasome system as a critical regulator of synaptic plasticity and long-term memory formation. Neurobiol Learn Mem.

[20] Karpova A, Mikhaylova M, Thomas U, Knopfel T, Behnisch T (2006). Involvement of protein synthesis and degradation in long-term potentiation of Schaffer collateral CA1 synapses. J Neurosci.

[21] Krueger F, Andrews SR (2011). Bismark: a flexible aligner and methylation caller for bisulfite-seq applications. Bioinformatics.

[22] Akalin A, Kormaksson M, Li S, Garrett-Bakelman FE, Figueroa ME, Melnick A (2012). methylKit: a comprehensive R package for the analysis of genome-wide DNA methylation profiles. Genome Biol.

[23] Benjamini Y, Hochberg Y (1995). Controlling the false discovery rate: a practical and powerful approach to multiple testing. J R Stat Soc Ser B (Methodol).

[24] Joseph A, Turrigiano GG (2017). All for one but not one for all: excitatory synaptic scaling and intrinsic excitability are coregulated by CaMKIV, whereas inhibitory synaptic scaling is under independent control. J Neurosci.

[25] Wayman GA, Tokumitsu H, Davare MA, Soderling TR (2011). Analysis of CaM-kinase signaling in cells. Cell Calcium.

[26] Sakai Y, Suetake I, Shinozaki F, Yamashina S, Tajima S (2004). Co-expression of de novo DNA methyltransferases Dnmt3a2 and Dnmt3L in gonocytes of mouse embryos. Gene Expr Patterns.

[27] Dieterich DC, Karpova A, Mikhaylova M, Zdobnova I, Konig I, Landwehr M (2008). Caldendrin-Jacob: a protein liaison that couples NMDA receptor signalling to the nucleus. PLoS Biol.

[28] Auberson YP, Allgeier H, Bischoff S, Lingenhoehl K, Moretti R, Schmutz M (2002). 5-Phosphonomethylquinoxalinediones as competitive NMDA receptor antagonists with a preference for the human 1A/2A, rather than 1A/2B receptor composition. Bioorg Med Chem Lett.

[29] Fenteany G, Standaert RF, Lane WS, Choi S, Corey EJ, Schreiber SL (1995). Inhibition of proteasome activities and subunit-specific amino-terminal threonine modification by lactacystin. Science.

[30] Lee DH, Goldberg AL (1998). Proteasome inhibitors: valuable new tools for cell biologists. Trends Cell Biol.

[31] Meng L, Mohan R, Kwok BH, Elofsson M, Sin N, Crews CM (1999). Epoxomicin, a potent and selective proteasome inhibitor, exhibits in vivo antiinflammatory activity. PNAS.

[32] Sarikas A, Hartmann T, Pan ZQ (2011). The cullin protein family. Genome Biol.

[33] Petroski MD, Deshaies RJ (2005). Function and regulation of cullin-RING ubiquitin ligases. Nat Rev Mol Cell Biol.

[34] Scudder SL, Patrick GN (2015). Synaptic structure and function are altered by the neddylation inhibitor MLN4924. Mol Cell Neurosci.

[35] Vogl AM, Brockmann MM, Giusti SA, Maccarrone G, Vercelli CA, Bauder CA (2015). Neddylation inhibition impairs spine development, destabilizes synapses and deteriorates cognition. Nat Neurosci.

[36] Soucy TA, Smith PG, Milhollen MA, Berger AJ, Gavin JM, Adhikari S (2009). An inhibitor of NEDD8-activating enzyme as a new approach to treat cancer. Nature.

[37] Brockmann MM, Dongi M, Einsfelder U, Korber N, Refojo D, Stein V (2019). Neddylation regulates excitatory synaptic transmission and plasticity. Sci Rep..

[38] Sakimura K, Kutsuwada T, Ito I, Manabe T, Takayama C, Kushiya E (1995). Reduced hippocampal LTP and spatial learning in mice lacking NMDA receptor epsilon 1 subunit. Nature.

[39] Kiyama Y, Manabe T, Sakimura K, Kawakami F, Mori H, Mishina M (1998). Increased thresholds for long-term potentiation and contextual learning in mice lacking the NMDA-type glutamate receptor epsilon1 subunit. J Neurosci.

[40] Karpova NN (2014). Role of BDNF epigenetics in activity-dependent neuronal plasticity. Neuropharmacology.

[41] Aid T, Kazantseva A, Piirsoo M, Palm K, Timmusk T (2007). Mouse and rat BDNF gene structure and expression revisited. J Neurosci Res.

[42] Zheng F, Zhou X, Luo Y, Xiao H, Wayman G, Wang H (2011). Regulation of brain-derived neurotrophic factor exon IV transcription through calcium responsive elements in cortical neurons. PloS One.

[43] Kundakovic M, Gudsnuk K, Herbstman JB, Tang D, Perera FP, Champagne FA (2015). DNA methylation of BDNF as a biomarker of early-life adversity. Proc. Natl Acad. Sci. USA.

[44] Maynard KR, Hill JL, Calcaterra NE, Palko ME, Kardian A, Paredes D (2016). Functional role of BDNF production from unique promoters in aggression and serotonin signaling. Neuropsychopharmacology.

[45] Assini FL, Duzzioni M, Takahashi RN (2009). Object location memory in mice: pharmacological validation and further evidence of hippocampal CA1 participation. Behav Brain Res.

[46] Haettig J, Sun Y, Wood MA, Xu X (2013). Cell-type specific inactivation of hippocampal CA1 disrupts location-dependent object recognition in the mouse. Learn Mem.

[47] Intlekofer KA, Berchtold NC, Malvaez M, Carlos AJ, McQuown SC, Cunningham MJ (2013). Exercise and sodium butyrate transform a subthreshold learning event into long-term memory via a brain-derived neurotrophic factor-dependent mechanism. Neuropsychopharmacology.

[48] Wang M, Li D, Yun D, Zhuang Y, Repunte-Canonigo V, Sanna PP (2017). Translation of BDNF-gene transcripts with short 3’ UTR in hippocampal CA1 neurons improves memory formation and enhances synaptic plasticity-relevant signaling pathways. Neurobiol Learn Mem.

[49] Wyllie DJ, Livesey MR, Hardingham GE (2013). Influence of GluN2 subunit identity on NMDA receptor function. Neuropharmacology.

[50] Zheleznyakova GY, Cao H, Schioth HB (2016). BDNF DNA methylation changes as a biomarker of psychiatric disorders: literature review and open access database analysis. Behav Brain Funct.

[51] Zhubi A, Veldic M, Puri NV, Kadriu B, Caruncho H, Loza I (2009). An upregulation of DNA-methyltransferase 1 and 3a expressed in telencephalic GABAergic neurons of schizophrenia patients is also detected in peripheral blood lymphocytes. Schizophr Res.

[52] Oliveira AM, Hemstedt TJ, Bading H (2012). Rescue of aging-associated decline in Dnmt3a2 expression restores cognitive abilities. Nat Neurosci.

[53] Oliveira AM, Hemstedt TJ, Freitag HE, Bading H (2016). Dnmt3a2: a hub for enhancing cognitive functions. Mol Psychiatry.

