# Supplementary material for: Synaptic control of DNA methylation involves activity-dependent degradation of DNMT3A1 in the nucleus
Source: Neuropsychopharmacology. 2020 Jul 29;45(12):2120–30. doi: 10.1038/s41386-020-0780-2 (PMC7547096; doi:10.1038/s41386-020-0780-2)
Supplement: Supplementary file 1 — Supplement [file 41386_2020_780_MOESM1_ESM.docx]

**Supplementary Figures**


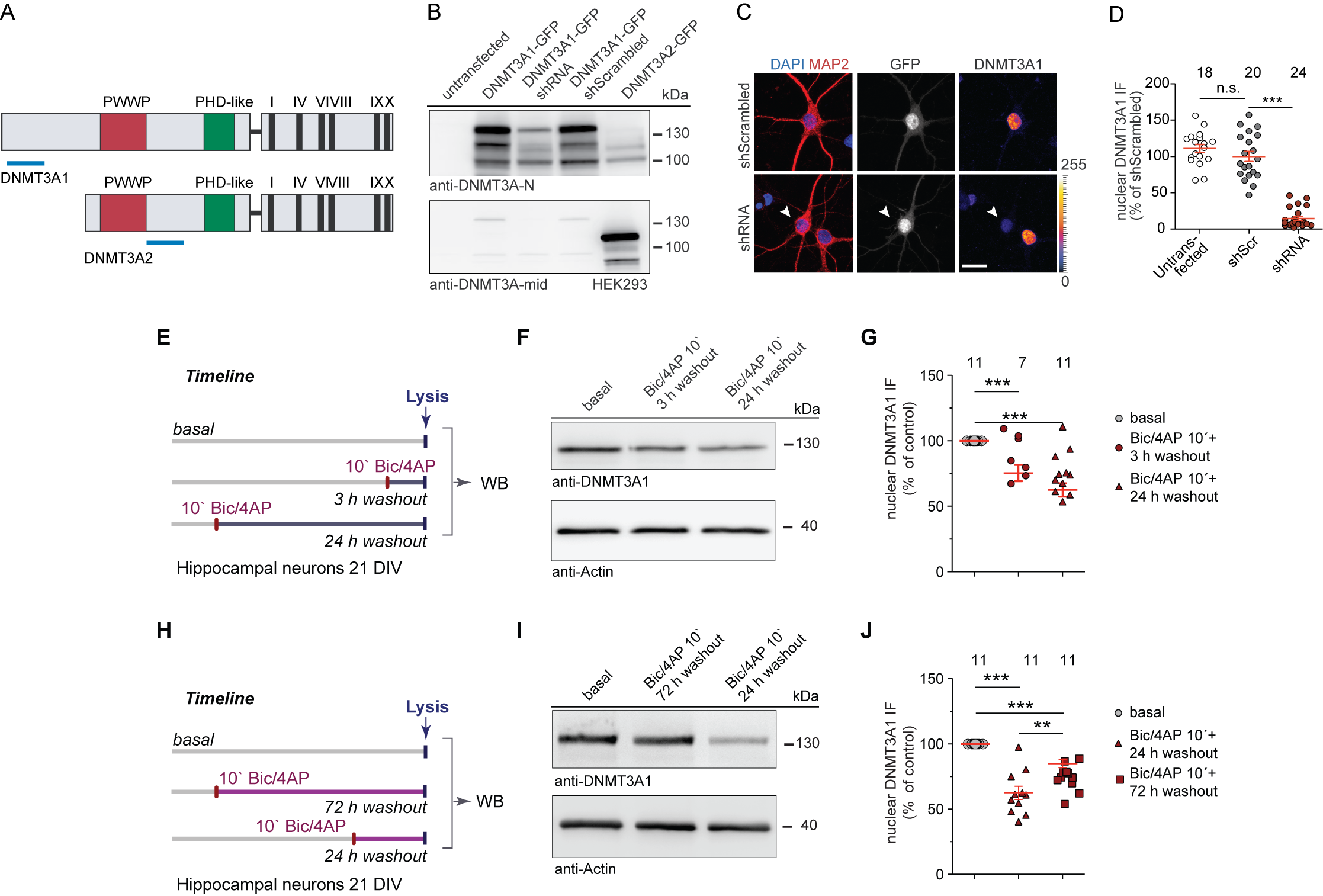


**Figure S1. Verification of the specificity of the DNMT3A isoform1 antibody and recovery of DNMT3A1 nuclear protein levels following synaptic stimulation.**

**A** The domain structure of the two *de novo* DNA methyltransferase DNMT3A1 and DNMT3A2 is depicted. The epitopes of the DNMT3AN and DNMT3A-mid antibody are indicated with blue bars.

**B** Heterologous expression of DNMT3A1 and DNMT3A2 and shRNA-based mRNA knockdown in HEK-T cells was detected with either the Dnmt3aN specific antibody (upper panel) or the DNMT3A-mid antibody recognizing both isoforms (lower panel). **C** and **D** Representative images and quantification depicts the nuclear DNMT3A1 isoform knockdown in 16 DIV primary hippocampal neurons. Unpaired two-tailed Student’s t-test, n.s. not significant, *** p<0.0001. Scale bar is 20 μm. Error bars present S.E.M. Sample numbers for each experimental group indicate neurons from three different culture preparations. **E-J** Time-dependent recovery of degraded pool of DNMT3A1 protein. Timeline view of the treatment of cultured hippocampal neurons with Bic/4AP for 10 min followed by **E** 3 hrs or 24 hrs washout and **H** 24 hrs or 72 hrs washout of antagonist-containing media. **F** and **I** Quantitative immunoblots show the degradation of DNMT3A1 3 hrs and 24 hrs after the neuronal activity triggered by a 10 min-stimulation. **I** and **J** The degraded pool of DNMT3A1 protein almost gets back to basal condition levels 72 hrs after the 10 min-long Bic/4AP treatment. One-sample t-test is performed while the hypothetical value is set to 100. **p<0.01, ***p<0.001. Error bars present S.E.M. Sample numbers for each experimental group indicate neurons from three different culture preparations.

**
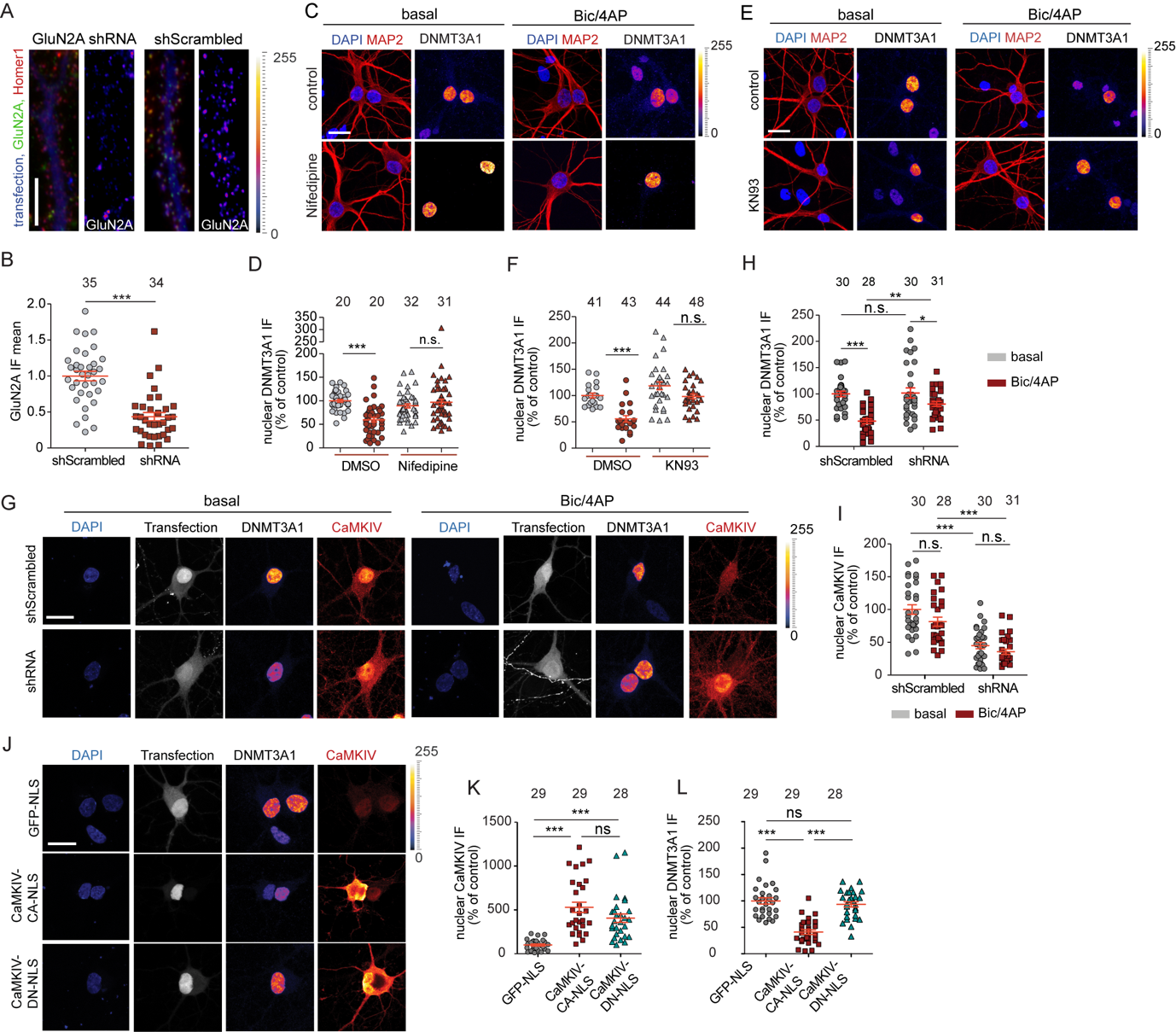
**

**Figure S2. DNMT3A1 degradation upon neuronal stimulation is CaMKIV-dependent.**

**A** and **B** Representative immunofluorescence images of 11 DIV hippocampal primary neurons transfected with either Scrambled or GluN2A shRNA, fixed after 4 days after transfection at 15 DIV. shRNA knockdown confirms the significant less surface expression of GluN2A. Scale bar is 10 μm. Unpaired two-tailed Student’s t-test.

**C** and **D** The L-type calcium channel blocker Nifedipine (10 μM) attenuated the degradation of DNMT3A1 when 14-15 DIV hippocampal primary neurons were stimulated by Bic/4AP for 6hrs. Two-way ANOVA followed by Bonferroni’s post hoc test. Scale bar is 20 μm.

**E** and **F** The CamKII and IV inhibitor KN93 (5 μM) abolished the degradation of DNMT3A1 when 14-15 DIV hippocampal primary neurons were stimulated by Bic/4AP for 6hrs. Scale bar is 20 μm. Two-way ANOVA followed by Bonferroni’s post hoc test. ***P<0.001, n.s. not significant.

**G** to **I** Representative immunofluorescence images and quantification of CaMKIV immunofluorescence in hippocampal primary neurons expressing Scrambled or CaMKIV shRNA Two-way ANOVA followed by Bonferroni’s post hoc test. Scale bar is 20 μm. ***P<0.001, n.s.: not significant.

**J** to **L** Representative immunofluorescence images (J) and quantification of nuclear CaMKIV (K) and DNMT3A1 (L) in hippocampal primary neurons overexpressing constitutively active or dominant negative CaMKIV. One-way ANOVA followed by Tukey`s post hoc test. Scale bar is 20 μm. ***P<0.001, NLS nuclear localization signal, n.s.: not significant. Error bars present S.E.M. Sample numbers for each experimental group indicate neurons from three different culture preparations.

**
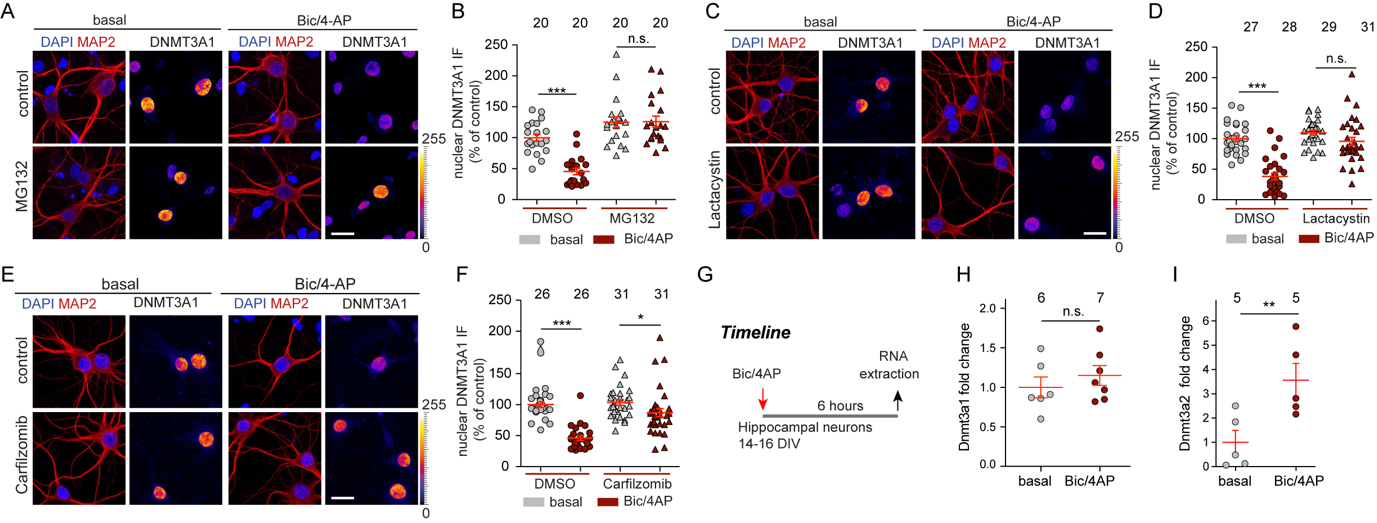
**

**Figure S3. Proteasomal degradation of DNMT3A1 following synaptic stimulation.**

**A** and **B** Inhibition of the proteasome with 30 μM MG132, **C** and **D** 15 μM Lactacystin and **E** and **F** 100 nM Carfilzomib prevented degradation of DNMT3A1 upon neuronal stimulation. Scale bars are 20 μm. Two-way ANOVA followed by Bonferroni’s post hoc test. ***P<0.001, n.s. not significant. **G** Timeline view of the qPCR experiments performed upon synaptic stimulation for *Dnmt3a1* and *2* transcripts. **H** The expression of *Dnmt3a1* transcript specific to DNMT3A1 protein expression was not altered following the treatment of 14-15 DIV hippocampal primary neurons with Bic/4AP for 6 hrs. Unpaired Student’s t-test, p=0.4229. **I** *Dnmt3a2* transcript is upregulated upon synaptic stimulation. Unpaired Student’s t-test, **p<0.01. Error bars present S.E.M. Sample numbers for each experimental group indicate neurons from three different culture preparations.


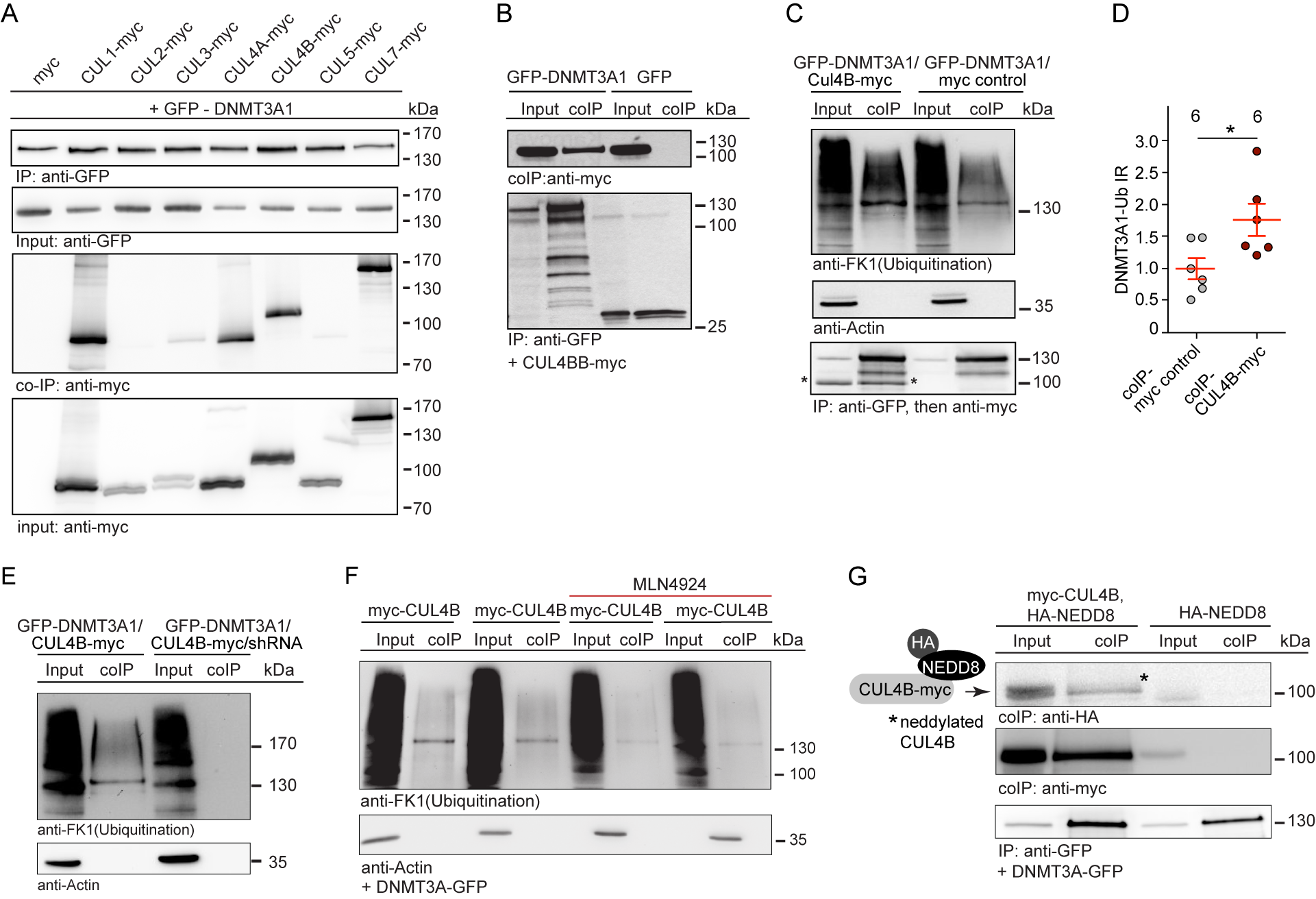


**Figure S4. Neddylation of the Cul4B enhances DNMT3A1 degradation.**

**A** Heterologous expression of GFP-DNMT3A1 and empty myc-vector or human myc-Cullins (CUL1, CUL2, CUL3, CUL4A, CUL4B, CUL5, CUL7) in HEK293-T cells. DNMT3A1 was immunoprecipitated from total cell extracts using anti-GFP antibodies coupled to microbeads. Cullin proteins were detected in the immunoprecipitate with a myc-antibody. The same volume of IP and input per sample were loaded.

**B** HEK-T cells were transfected with expression vectors for GFP-DNMT3A1 and myc-CUL4B or myc-vector. DNMT3A1 was immunoprecipitated from total cell extracts using anti-GFP antibodies coupled to microbeads. CUL4B was detected in the immunoprecipitate with a myc-antibody.

**C** and **D** Poly-ubiquitination of DNMT3A1 in the immunoprecipitate is enhanced when DNMT3A1 was co-expressed with CUL4B. Unpaired two-tailed Student’s t-test, *p<0.05.

**E** shRNA knockdown of CUL4B reduces polyubiquitination of DNMT3A1.

**F** and **G** HEK-T cells were transfected with expression vectors for GFP-DNMT3A1, myc-CUL4B (**F**) and also with active HA-Nedd8 (**G**). Total cell extracts were immunoprecipitated using anti-GFP antibodies. (**F**) Less DNMT3A1 ubiquitination was observed when the NAE inhibitor MLN4924 (94nM) was applied for 24hrs. (**G**) Neddylated CUL4B was detected with anti-HA antibody in the DNMT3A1 elute. Error bars present S.E.M. Sample numbers for each experimental group indicate neurons from at least two different culture preparations.

**
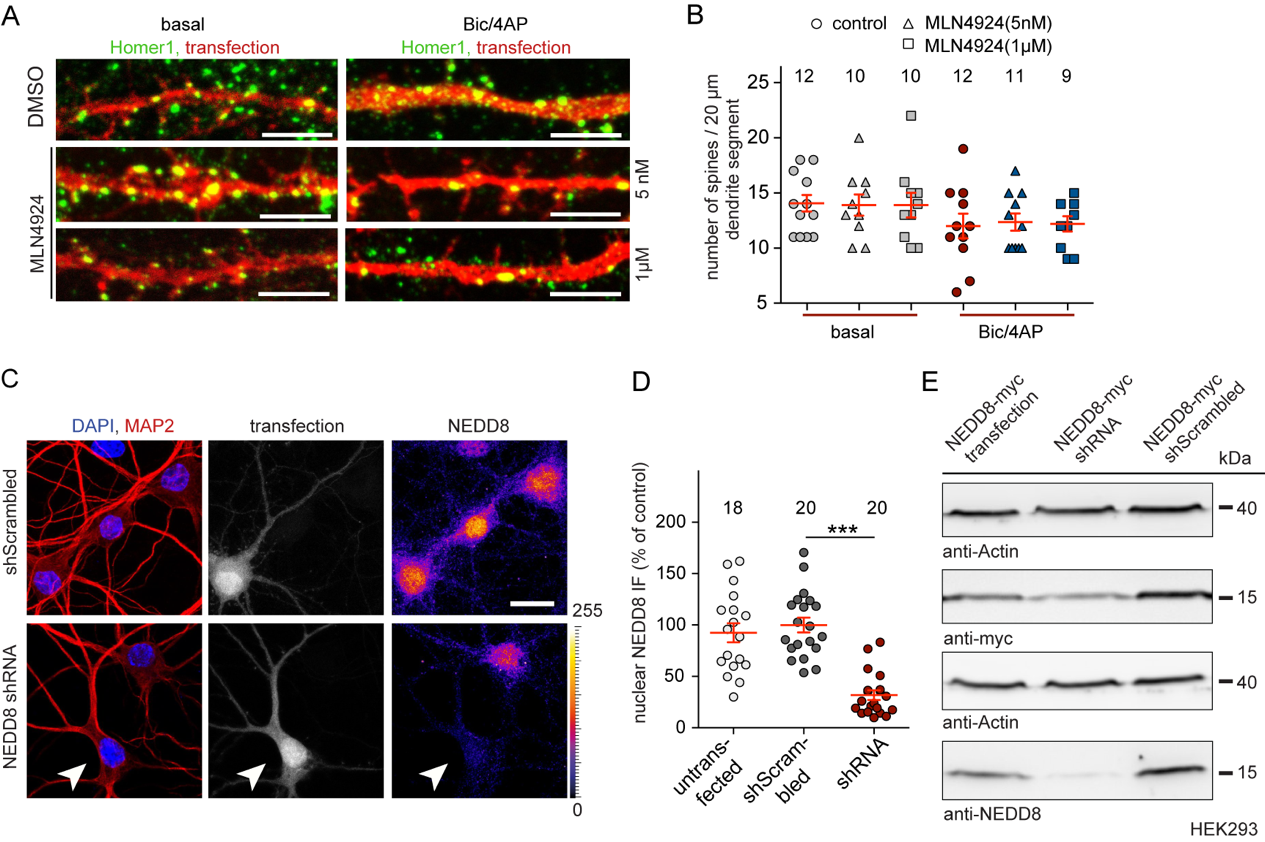
**

**Figure S5. Inhibition of neddylation does not alter total spine number.**

**A** and **B** DIV 14 hippocampal neurons were transfected with RFP expressed under control of an actin promoter and cells were fixed two days after transfection. Homer1 positive dendritic spine numbers were counted. There is not any significant alteration in the total spine number following MLN4924 treatments following the synaptic stimulation with Bic/4AP for 6 hrs. Scale bars are 5 μm.

**C** and **D** Representative images and quantification display the endogenous rat-Nedd8 knockdown generated in 16 DIV primary hippocampal neurons. Unpaired two-tailed Student’s t-test, *** p<0.0001. Scale bar in C is 20 μm.

**E** Heterologous expression of human-NEDD8 and shRNA mRNA knockdown and shScrambled control in HEK-T cells was detected with either the anti-myc antibody (upper panel) or the anti-NEDD8 antibody (lower panel). The knockdown of Nedd8 was detected by anti-myc and as well as anti-NEDD8 antibodies. Error bars present S.E.M. Sample numbers for each experimental group indicate neurons from three different culture preparations.


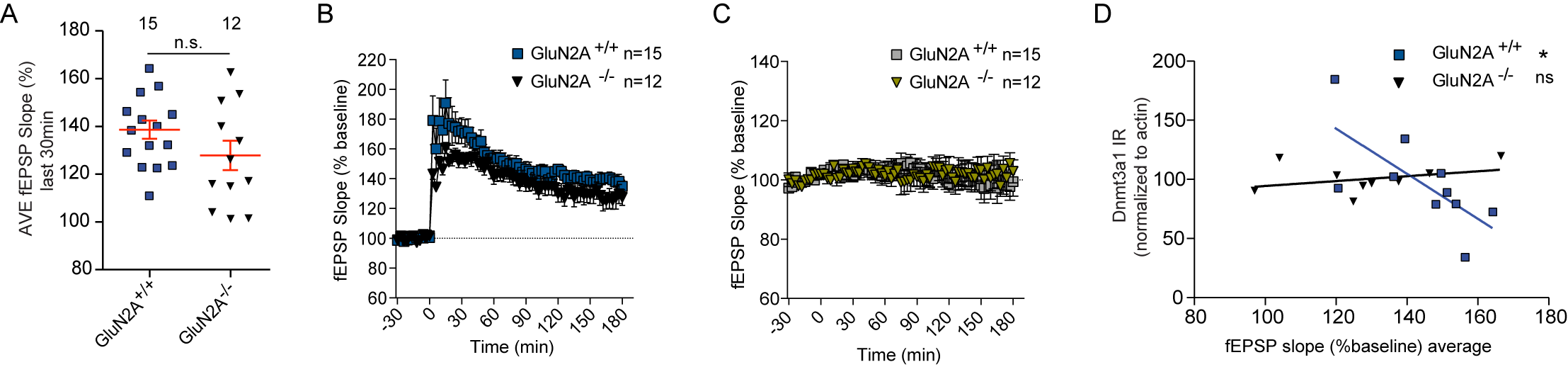


**Figure S6. Recovered LTP in GluN2A knock-out mice.**

**A** Averaged fEPSP slopes of the last 30 min LTP showed no significant difference between GluN2A+/+ and GluN2A-/- mice. The Mann Whitney Test was used to compare the Averaged field potentials (150-180min) between two groups.

**B** LTP induced by HFS (3 trains high frequency stimulus @100Hz) in GluN2A-/- mice (n=12 slices from 6 animals) was similar to GluN2A+/+ mice (n=15 slices from 6 animals).

**C** Baseline was determined by the second input on the same slice which was induced LTP by the first input.

**D** Scatterplot and correlation analysis between the normalized DNMT3A1 immunoblotting mean values and the averaged fEPSP slope of the whole LTP recording depicts a negative correlation between the strength of the LTP and the synaptic stimulation-induced DNMT3A1 down-regulation in GluN2A +/+ mice while no significant correlation was observed in slices of GluN2A -/- mice. Unpaired two-tailed Student’s t-test, *p<0.05, ns: not significant. Error bars present S.E.M. Sample numbers for each experimental group indicate mice used per group.

**
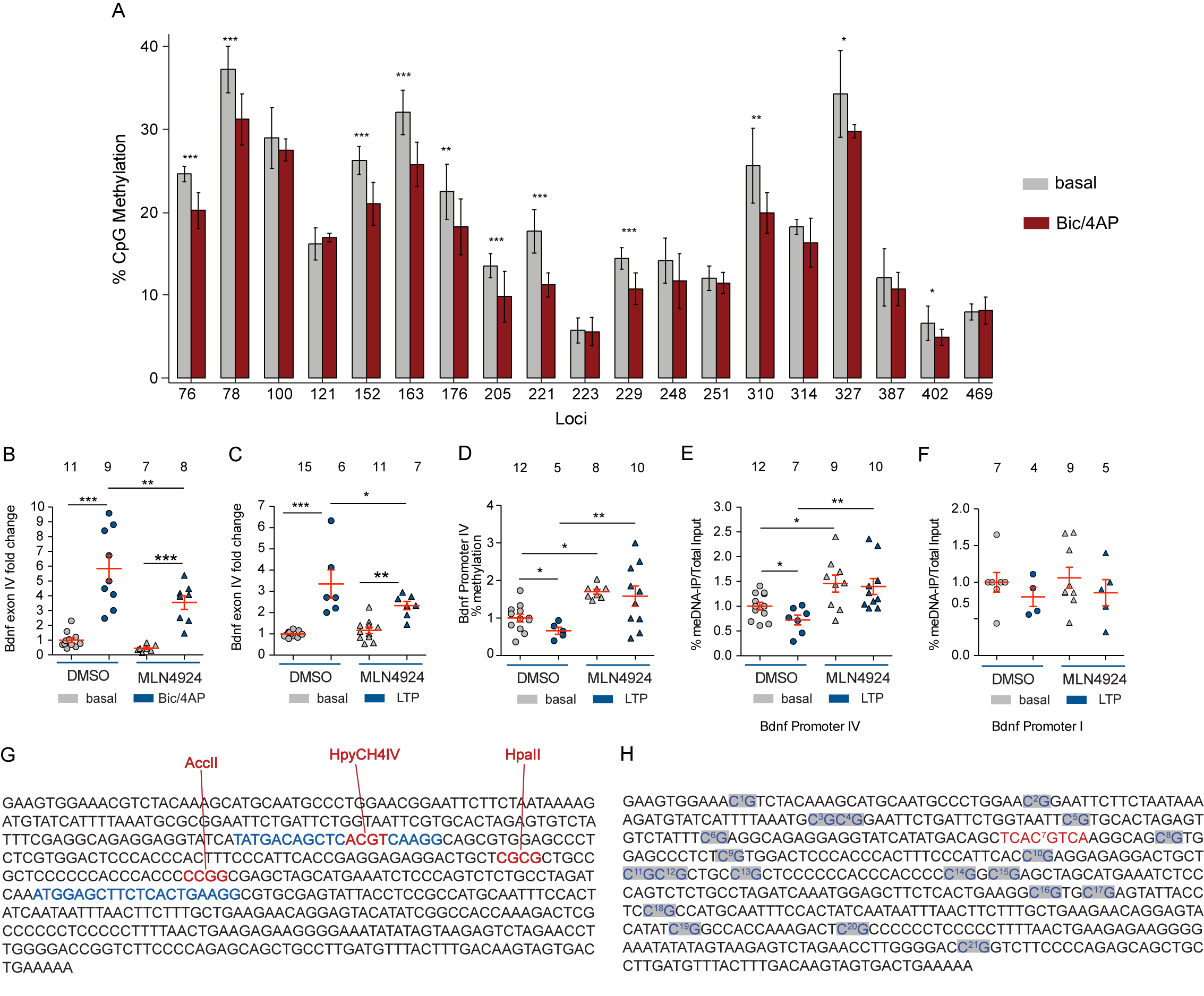
**

**Figure S7. *Bdnf IV* gene expression and promoter methylation is regulated by neddylation in neurons upon synaptic stimulation.**

**A** Removal of tonic inhibition by Bic/4AP treatment for 6 hrs in cultured neurons led to demethylation of the majority of the CpGs in the *Bdnf* IV promoter. Data shown with S.E.M. The p-value was obtained using a Chi-square test and corrected using FDR *p < 0.05, **p < 0.01, ***p < 0.001.

**B** Bdnf exon IV expression was induced in cultured hippocampal primary neurons following Bic/4AP treatment for 6hrs and this induction was lowered by 5 nM MLN4924. Two-way ANOVA followed by Bonferroni’s post hoc test, **p<0.01, ***p<0.001.

**C** Bdnf exon IV expression was induced following LTP induction for 6hrs in the CA1 region of Hippocampus and this induction was lowered by 50 nM MLN4924. Two-way ANOVA followed by Bonferroni’s post hoc test, *p<0.05 **p<0.01 ***p<0.001.

**D** Methylation analysis based on the restriction of the methylation-sensitive sites showed demethylation of Bdnf promoter IV following LTP induction in CA1. Unpaired one-tailed Student’s t- test, t15=1.939, p=0.0358; promoter methylation was increased when treated with MLN4924 irrespective of LTP induction or basal conditions. Two-way ANOVA followed by Bonferroni’s post hoc test, *p<0.05 **p<0.01.

**E** MeDIP-qPCR analysis revealed demethylation of Bdnf promoter IV following LTP induction in CA1 (Unpaired one-tailed Student’s t-test, t17=2,198 p=0.0421) and promoter methylation was increased when treated with MLN4924 irrespective of LTP induction or basal conditions. Two-way ANOVA followed by Bonferroni’s post hoc test, *p<0.05, **p<0.01.

**F** MeDIP-qPCR analysis did not show any alteration in Bdnf promoter I methylation. Two-way ANOVA followed by Bonferroni’s post hoc test.

**G** *Rattus norvegicus* Bdnf IV promoter sequence (chr3:100,786,676-100,787,200 on the UCSC genome browser) shows: Primers, highlighted in blue, used in the study spanning a sequence, which contains three methylation-sensitive restriction sites, depicted in red. *Rattus norvegicus* Bdnf IV promoter sequence (chr3:100,786,676-100,787,200 on the UCSC genome browser) shows: CG dinucleotides highlighted in grey and CRE binding site in red.

**H** *Rattus norvegicus* Bdnf IV promoter sequence (chr3:100,786,676-100,787,200 on the UCSC genome browser) shows: CG dinucleotides highlighted in grey and CRE binding site in red. Error bars present S.E.M. Sample numbers for each experimental group indicate neurons from three different culture preparations.
